# Aquaporin 1 confers apoptosis resistance in pulmonary arterial smooth muscle cells from the SU5416 hypoxia rat model

**DOI:** 10.14814/phy2.16156

**Published:** 2024-08-22

**Authors:** Xin Yun, Shannon Niedermeyer, Manuella Ribas Andrade, Haiyang Jiang, Karthik Suresh, Todd Kolb, Mahendra Damarla, Larissa A. Shimoda

**Affiliations:** ^1^ Division of Pulmonary and Critical Care Medicine Johns Hopkins School of Medicine Baltimore Maryland USA

**Keywords:** cell death, lung, pulmonary hypertension, remodeling

## Abstract

Pulmonary hypertension (PH) arises from increased pulmonary vascular resistance due to contraction and remodeling of the pulmonary arteries. The structural changes include thickening of the smooth muscle layer from increased proliferation and resistance to apoptosis. The mechanisms underlying apoptosis resistance in PH are not fully understood. In cancer cells, high expression of aquaporin 1 (AQP1), a water channel, is associated with apoptosis resistance. We showed AQP1 protein was expressed in pulmonary arterial smooth muscle cells (PASMCs) and upregulated in preclinical PH models. In this study, we used PASMCs isolated from control male rats and the SU5416 plus hypoxia (SuHx) model to test the role of AQP1 in modulating susceptibility to apoptosis. We found the elevated level of AQP1 in PASMCs from SuHx rats was necessary for resistance to apoptosis and that apoptosis resistance could be conferred by increasing AQP1 in control PASMCs. In exploring the downstream pathways involved, we found AQP1 levels influence the expression of Bcl‐2, with enhanced AQP1 levels corresponding to increased Bcl‐2 expression, reducing the ratio of BAX to Bcl‐2, consistent with apoptosis resistance. These results provide a mechanism by which AQP1 can regulate PASMC fate.

## 1 INTRODUCTION

Pulmonary arterial hypertension (PAH) is a life‐threatening condition that can arise from various etiologies. Regardless of the inciting cause, increased pulmonary vascular resistance due to contraction and remodeling of the pulmonary arteries leads to increased afterload on the right ventricle, ultimately resulting in right ventricular failure. Currently approved therapies provide symptom relief and can slow disease progression by inducing vasodilation, but none specifically target the structural remodeling that is a key feature of PAH. Thus, there are no curative therapeutic options.

The vascular remodeling observed in both patients with PAH and animal models includes intimal and medial thickening due to cell proliferation, extension of muscle into nonmuscular precapillary arterioles, likely due to migration of pulmonary arterial smooth muscle cells (PASMCs) and endothelial‐to‐mesenchymal transdifferentiation (Jurasz et al., 2010; Masri et al., 2007; Morrell et al., 2001; Yu et al., 2004). While these processes are prevalent in the early stages of disease and contribute to vascular narrowing, it is increasingly recognized that as the condition progresses, resistance to apoptosis and failure of cell turnover becomes a significant contributor (Lampron et al., 2020; Meloche et al., 2015; Stenmark et al., 2018). Normally, apoptosis is a highly regulated process of cell death that maintains homeostasis by orderly removal of nonfunctioning or damaged cells. Impaired apoptosis, as observed in many types of cancer cells (Hua et al., 2023; Lee et al., 2023), leads to uncontrolled proliferation and tumor growth. In the case of PAH, resistance to apoptosis has been reported in PASMCs from patients (Dromparis et al., 2010; Lampron et al., 2020; Meloche et al., 2015) and is believed to contribute to vascular narrowing and/or complete obliteration of the arterial lumen.

The mechanisms underlying apoptosis resistance in PAH are not fully understood. In cancer cells, several reports linked excess expression of the protein, aquaporin 1 (AQP1), with apoptosis resistance (Ding et al., 2013; Hoque et al., 2006; Simone et al., 2018; Wu et al., 2015; Zhang et al., 2019). AQP1 is a water channel that is expressed in a variety of cell types and tissues, including red blood cells, endothelium, renal tubules, tumor cells, and sweat glands (Agre et al., 2002). We showed functional AQP1 protein was expressed in PASMCs (Leggett et al., 2012) and was upregulated in preclinical models of PH, including the chronic hypoxia (Leggett et al., 2012) and SU5416 plus hypoxia (SuHx) (Yun et al., 2021) rat models. Loss of AQP1 attenuated the development of PH in response to chronic hypoxia (Liu et al., 2019), and we showed AQP1 was required for PASMC migration and proliferation via a mechanism involving the C‐terminal tail and upregulation of β‐catenin (Lai et al., 2014; Yun et al., 2017). Whether AQP1 controls susceptibility of PASMCs to apoptosis in the SuHx model is unknown, although in unstimulated control human PASMCs (Schuoler et al., 2017) and in murine PASMCs subjected to in vitro hypoxia (Liu et al., 2019), loss of AQP1 increased caspase 3/7 activity, which could be consistent with increasing apoptosis.

The mechanism by which AQP1 might regulate apoptosis is unclear. When cells are subjected to stress, via mitochondrial dysfunction, oxidative damage, stimulated proliferation, or exposure to apoptotic stimuli, a series of well‐characterized events are set into motion, including suppression of Bcl‐2, an inhibitor of the pro‐apoptotic molecule, Bcl‐2‐like protein 4 (BAX) (Edlich, 2018). Upon activation, BAX homo‐oligomerizes and translocates to, and permeabilizes, the mitochondrial membrane, allowing release of cytochrome C (cyt C) from damaged mitochondria (Moldoveanu & Czabotar, 2020). Cytosolic cyt C then activates caspase 3, resulting in DNA fragmentation and condensation, cell blebbing, and formation of apoptotic bodies disposed of through phagocytosis. In cancer cells, AQP1 has been proposed to be upstream of the Bcl‐2 pathway (Shu et al., 2019; Zhang et al., 2018). Whether Bcl‐2 and BAX expression are regulated by AQP1 in PASMCs is unknown.

In this study, we tested the hypothesis that enhanced AQP1 expression in PASMCs from the well‐established SuHx rat model of PAH contributes to apoptosis resistance due to reciprocal regulation of BAX and Bcl‐2, with high AQP1 levels causing an increase in Bcl‐2 and repression of BAX. We tested this hypothesis using gain and loss of function experiments in PASMCs isolated from normoxic control rats and from the SuHx model.

## 2 METHODS

All protocols were reviewed by and performed in accordance with the Johns Hopkins University Animal Care and Use Committee (protocol #RA23M141). Protocols and procedures comply with NIH and Johns Hopkins Guidelines for the care and use of laboratory animals.

### 2.1 Sugen/hypoxia model

**FIGURE 1:**
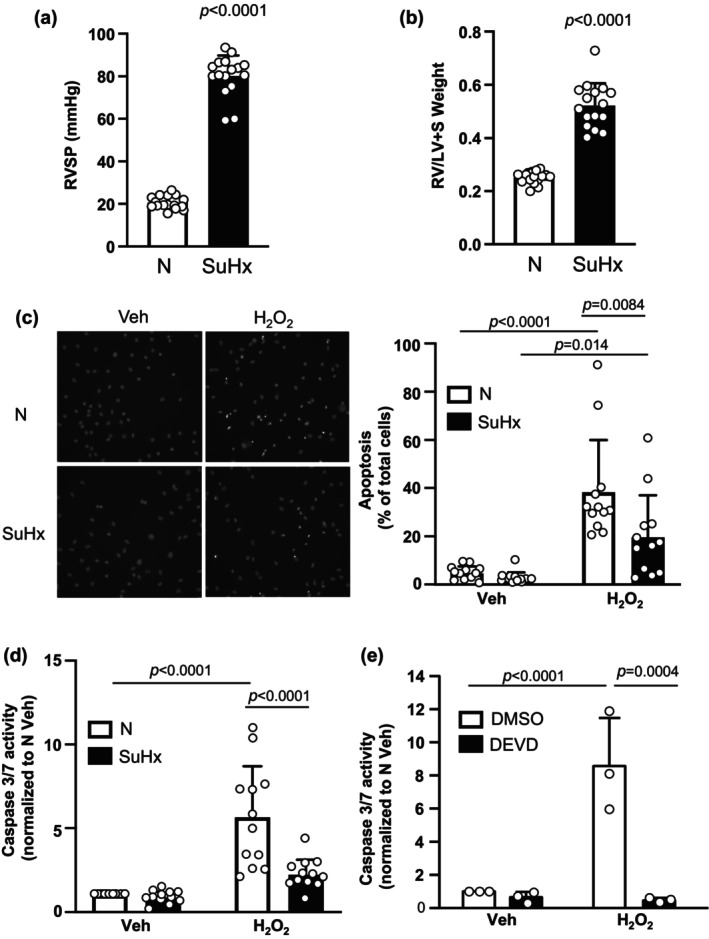
Apoptosis in pulmonary arterial smooth muscle cells (PASMCs) from normoxic (N) and Su5416+hypoxia (SuHx) rats. (a, b) Bar and scatter graphs represent mean ± SD values and individual values (*n* = 16 rats for each group) for (a) right ventricular systolic pressure (RVSP) and (b) right ventricle to left ventricle plus septum (RV/LV+S) weight in N and SuHx rats. Significance was determined by *t* test. (c, d) Bar and scatter graphs represent mean ± SD values and individual values (*n* = 12 rats per group) for apoptosis measured via (c) Hoescht staining and (d) caspase 3/7 activity assay in PASMCs from N and SuHx rats challenged with vehicle (Veh; PBS) or H_2_O_2_ (500 μM; 24 h). Values are presented as percent of total cells. Significance assessed by two‐way anova with Holm‐Sidak post hoc test. For (c), interaction *p* = 0.045. For (d), interaction *p* = 0.001. (e) Bar and scatter graphs represent mean ± SD values and individual values (*n* = 3 per group) for caspase 3/7 activity in PASMCs from N rats challenged with Veh or H_2_O_2_ in the presence of DMSO (vehicle) or the caspase 3/7 inhibitor DEVD (50 mg/mL). Significance assessed by two‐way anova with Holm‐Sidak post hoc test. Interaction *p* = 0.0077.

Pulmonary hypertension was induced in adult male Wistar rats (150–200 g; Harlan Farms) by a single subcutaneous injection of SU5416 (20 mg/kg, Tocris, Cat. No. 3037), prepared in a carboxymethylcellulose (CMC)‐containing diluent as described previously (Huetsch et al., 2016), followed by exposure to 10% O_2_ (hypoxia) for 3 weeks. The rats were exposed to normoxic conditions for <5 min twice a week to change cages and replenish food (Regular chow, PMI Nutrition International LLC) and water. At the end of 3 weeks, rats were returned to normoxia for an additional 2 weeks. Control rats were injected with vehicle and maintained in room air (normoxia) for 5 weeks. All animals were kept in the same room and exposed to the same light–dark cycle and ambient temperature and were housed in standard rat cages (3 rats/cage) with free access to food and water. At the end of exposures, rats were anesthetized (Ketamine, 75 mg/kg; Xylazine, 7.5 mg/kg) and depth of anesthesia was confirmed via paw pinch prior to measurement of right ventricular systolic pressure (RVSP) via transdiaphragmatic right heart puncture with a heparinized 23 gauge needle attached to a pressure transducer for 1–3 min. Right ventricular systolic pressure for each animal was measured as the average of at least 5 continuous heartbeats in animals with a minimum heart rate of >200 bpm. Exposure to the SuHx protocol resulted in an increase in RVSP (Figure 1a). Following measurement of RVSP, animals were euthanized via exsanguination and the heart and lungs removed and transferred to a dissecting dish filled with cold N‐[2‐hydroxyethyl]piperazine‐N′‐[2‐ethanesulfonic acid] HEPES‐buffered salt solution (HBSS) containing (in mmol/L): 130 NaCl, 5 KCl, 1.2 MgCl_2_, 1.5 CaCl_2_, 10 HEPES, and 10 glucose, with pH adjusted to 7.2 with 5 mol/L NaOH (all chemicals from Sigma Aldrich). Under a microscope, the atria and large conduit vessels were removed and the right ventricle (RV) wall was carefully separated from the left ventricle and the septum (LV+S). Both portions were blotted dry and weighed. As shown in Figure 1b, RV/LV+S weight ratio was greater in SuHx rats compared to normoxic rats. Coupled with the increase in RVSP in SuHx rats, these results indicate development of pulmonary hypertension in our model.

### 2.2 Isolation and culture of PASMCs

Resistance‐level pulmonary arteries (200–600 μM outer diameter) were isolated, the adventitia removed and arteries opened for endothelial denudation with a cotton swab. Tissue was placed in 4°C HBSS for at least 30 min, transferred to room temperature reduced‐Ca^2+^ HBSS (20 μm CaCl_2_) for at least 20 min and then digested in reduced‐Ca^2+^ HBSS containing: type I collagenase (1750 U/mL; gibco, #17100‐017), papain (9.5 U/mL; Sigma Aldrich, #p4762), bovine serum albumin (2 mg/mL; Sigma Aldrich, #9048‐46), and DTT (1 mM; Sigma Aldrich, #3483‐12) at 37°C for 15–20 min. After digestion, the tissue was transferred to Ca^2+^‐free HBSS in a small round bottom tube and slowly pipetted up and down to create a single‐cell suspension. Dispersed PASMCs were cultured in a Smooth Muscle Cell Medium (Sciencell, Cat 1101) supplemented with smooth muscle cell bullet kit (Lonza; CC‐3182) with 1% penicillin–streptomycin to expand cell numbers. All cells were used at passage 1‐2 and were growth arrested in smooth muscle basal media (Lonza; CC‐3181) supplemented with 0.3% FBS for 24–48 h before beginning experiments. Assessment of smooth muscle culture purity was performed using (1) [Ca^2+^]_i_ responses to 80 mM KCl and (2) immunofluorescence in cells stained with smooth muscle‐specific α‐actin (SMA; 1:400, A2547, Sigma) and calponin (1:100, ab700, Abcam) or smooth muscle myosin heavy chain (SMMHC; 1:100, ab125884, Abcam) and DAPI (nuclear stain; 1:10,000 in PBS, Invitrogen, R37606). Only cultures where >90% of cells exhibited at least 50 nM increase in [Ca^2+^]_i_ and/or positive SMA and calponin or SMMHC expression were used for these studies.

### 2.3 Hydrogen peroxide (H_2_O_2_ ) exposures

To induce apoptosis, cells were exposed to H_2_O_2_ (Sigma). At approximately 60% confluence, cells were placed into basal media for 24 h. Cells were then washed three times with PBS before being placed in serum‐free media containing freshly made H_2_O_2_ (500 μM) and incubated for 24 h before additional measurements (apoptosis, caspase activity, or immunoblot) were made.

### 2.4 Hoechst staining

Pulmonary arterial smooth muscle cells were stained with Hoechst 33342 dye (3 μL/mL of 1:100 dilution; 62249; Invitrogen, R37605) for 15–30 min at 37°C to visualize apoptotic cells. Apoptosis was identified by observing alterations in chromatin morphology (i.e., condensation) upon staining. For each sample, nine images were captured at 20× via fluorescent microscopy and analyzed using ImageJ. Apoptosis was determined as the percent of apoptotic cells over total cells and counts for each image within a sample were averaged to obtain a single value.

### 2.5 Caspase activity assay

Caspase 3/7 activity was measured using the Caspase‐Glo® 3/7 Assay System (G8090, Promega) per the manufacturer's instructions. Briefly, equal amounts of cell lysate (2.5 or 5 μg) and T‐PER (ThermoFisher, 78510) for each sample were placed into the wells of a 96‐well plate in duplicate. Proluminescent caspase 3/7 DEVD‐aminoluciferin substrate was added to each well and read with a luminometer at 5‐min intervals up to 60 min. Luminescence values within the linear portion of the curve (typically at 30 min) were background subtracted and normalized to control.

### 2.6 Western blotting

Pulmonary arterial smooth muscle cells were washed with PBS, and total protein was extracted in ice‐cold T‐PER buffer containing protease inhibitors (Roche Diagnostics, 4693132001). Proteins were quantified by use of the BCA protein assay (Pierce, 23225), and 5 μg total protein was resolved by 10% SDS‐PAGE gels transferred onto polyvinylidene difluoride membranes, which were then blocked with 5% nonfat dry milk in Tris‐buffered saline containing 0.2% Tween 20. Membranes were probed with primary antibodies AQP1 at 1:4000 (AQP11A, Alpha Diagnostic Intl. Inc.), Bcl‐2 at 1:1000 (ab196495, Abcam), or BAX at 1:1000 (ab32503, Abcam). Antibodies are verified with siRNA knockout by our lab previously (Leggett et al., 2012) or the antibody company. Bound antibodies were probed with horseradish peroxidase‐conjugated anti‐rabbit or anti‐mouse IgG (1:10,000, 52200336 and 52200341, Kirkegaard & Perry Laboratories) and detected by enhanced chemiluminescence (Clarity Western ECL Substrate, 170‐5061) using a Chemidoc (BioRad) gel imaging system. Membranes were then stripped and re‐probed for β‐tubulin (1:10,000, T7816, Sigma) as a housekeeping protein. Protein levels were quantified by densitometry using ImageJ.

### 2.7 Adenovirus infection

Adenoviral constructs containing a hemagglutinin (HA)‐tagged wild‐type AQP1 (AdAQP1), and GFP were created as described previously (Lai et al., 2014). Pulmonary arterial smooth muscle cells were placed in basal media and infected with virus (50 ifu/cell) for 48 h before being used in protein expression measurements and functional assays. Cells infected with the same adenovirus containing GFP (50 ifu/cell) were used as controls. We previously demonstrated expression and appropriate localization of AQP1 expressed using this adenovirus (Lai et al., 2014).

### 2.8 siRNA

Depletion of endogenous AQP1 was achieved using siRNA specifically targeting AQP1 (siAQP1; Horizon Discovery) and nontargeting (siNT; control) siRNA obtained as a “smart pool” (Horizon Discovery, D‐001810‐10‐50) as previously described (Leggett et al., 2012). Pulmonary arterial smooth muscle cells were incubated with 100 nM of siRNA for 16 h in serum‐ and antibiotic‐free media, after which serum was added to the media for a total concentration of 0.3% FBS. Cells were incubated under these conditions for 24 h, and then media was replaced and cells were incubated for an additional 48 h in basal media (0.3% FBS) prior to experiments.

### 2.9 Statistical analysis

Data are expressed as scatter plots with bars representing means ± SD. Each dot represents a separate experimental run, and since all experimental runs were performed on tissue/cells from different animals, “n” also refers to the number of animals. All data were tested for normality and equal variance prior to running statistical tests. Data that were not normally distributed were log (10) transformed and retested for normality and equal variance prior to running statistics. Statistical comparisons were performed using Student's *t* test for data in two groups or one‐ or two‐way anova with a Holm‐Sidak post hoc test for multiple‐group comparisons.

## 3 RESULTS

### 3.1 Effect of hydrogen peroxide on PASMCs

In cells isolated from normoxic rats, basal rates of apoptosis, measured via Hoescht staining, averaged less than 5% (Figure 1c). The average rate of basal apoptosis in PASMCs from SuHx rats was lower than, but not statistically different from, that measured in PASMC from normoxic rats. With application of the apoptotic stimulus, H_2_O_2_ (500 μM) for 24 h, the rate of apoptosis increased in normoxic cells to approximately 40%. In PASMCs from SuHx rats, the increase in apoptosis induced by H_2_O_2_ was significantly less (approximately 20%) than that observed in normoxic cells, indicating that SuHx cells have reduced susceptibility to apoptotic stimuli.

To complement the results obtained using Hoescht staining, we also performed caspase 3/7 activity assays. Consistent with our results with Hoechst staining, basal caspase 3/7 activity in unstimulated cells was not different between PASMCs from normoxic and SuHx rats (Figure 1d). As expected, challenge with H_2_O_2_ (500 μM) for 24 h resulted in a marked increase in caspase 3/7 activity in normoxic cells. In SuHx cells, caspase 3/7 activity after exposure to H_2_O_2_ was significantly lower than that observed in H_2_O_2_‐exposed normoxic cells and not significantly different from SuHx PASMCs under unstimulated conditions.

To verify the caspase 3/7 activity measures using the Caspase‐Glo assay truly reflected caspase 3/7 activity, we tested the effect of the specific inhibitor of caspase 3/7, DEVD. We repeated H_2_O_2_ exposure in cells from normoxic animals in the presence of DEVD (50 mg/mL; Cayman Chemical, #14414) or vehicle (DMSO) (Figure 1e). The addition of DEVD significantly reduced caspase 3/7 activity, confirming the luminescence measured was indeed due to activity of caspase 3/7.

### 3.2 Role of increased AQP1 protein in apoptosis susceptibility

**FIGURE 2:**
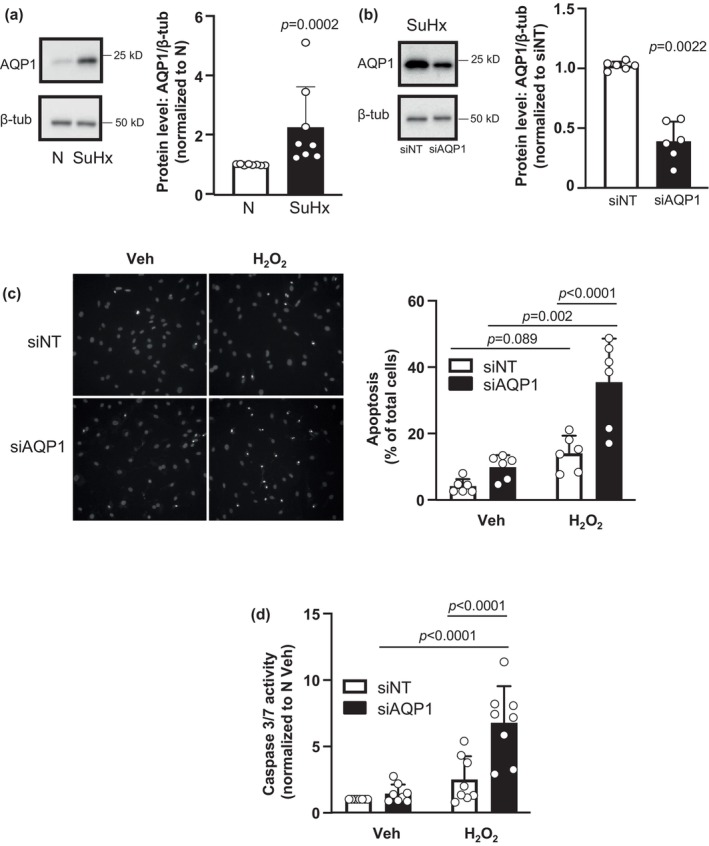
Effect of AQP1 depletion on apoptosis in pulmonary arterial smooth muscle cells (PASMCs). (a) Representative blots show AQP1 protein levels in PASMCs from normoxic (N) and Su5416/hypoxia (SuHx) rats. Bar and scatter plot graphs represent mean ± SD values and individual values (*n* = 8 rats per group) for AQP1 protein levels. Significance determined by *t* test. (b) Representative blots show siRNA targeted to AQP1 (siAQP1) reduced AQP1 protein levels in PASMCs from SuHx rats compared to cells transfected with a nontargeting siRNA (siNT). Bar and scatter plot graphs represent mean ± SD values and individual values (*n* = 6 rats per group) for AQP1 protein levels. Significance determined by *t* test. (c, d) Bar and scatter graphs represent mean ± SD values and individual values for apoptosis measured via (c) Hoescht staining (*n* = 6 rats per group) and (d) caspase 3/7 activity assay (*n* = 8 rats per group) in PASMCs from SuHx rats transfected with siNT or siAQP1 and challenged with vehicle (Veh; PBS) or H_2_O_2_ (500 μM; 24 h). Values for apoptosis are presented as percent of total cells while values for caspase 3/7 activity are normalized to siNT Veh cells. Significance assessed by two‐way anova with Holm‐Sidak post hoc test. For (c), interaction *p* = 0.0169. For (d), interaction *p* = 0.003.

We found that PASMCs from our SuHx rats exhibited increased AQP1 protein levels compared to controls (Figure 2a), consistent with our previously published results (Yun et al., 2021). Given the inverse correlation between AQP1 protein abundance and apoptosis reported in several tumor cell types (Lehnerdt et al., 2015; Moosavi & Elham, 2020; Simone et al., 2018; Tomita et al., 2017), we tested whether elevated levels of AQP1 could contribute to apoptosis resistance in SuHx PASMCs. Compared to cells treated with nontargeting siRNA (siNT), knockdown of AQP1 using siRNA (siAQP1) significantly reduced, but did not fully deplete, AQP1 protein in SuHx cells (Figure 2b). With knockdown of AQP1, the percent of cells from SuHx rats undergoing apoptosis at baseline (vehicle‐treated cells) was greater than in cells treated with siNT, although the difference did not reach statistical significance (*p* = 0.3431; Figure 2c). In response to H_2_O_2_, SuHx PASMCs treated with siNT were resistant to apoptosis, with a small increase in the percent of apoptotic cells that did not reach statistical significance compared to vehicle treatment. In contrast, depletion of AQP1 restored the ability of H_2_O_2_ to induce apoptosis in SuHx cells (Figure 2c). As shown in Figure 1, H_2_O_2_ has little effect on caspase 3/7 activity in SuHx cells. Consistent with our apoptosis results, caspase 3/7 activity in response to H_2_O_2_ was also significantly increased when AQP1 was depleted in SuHx cells (Figure 2d).

### 3.3 Effect of increasing AQP1 expression on apoptosis susceptibility in control PASMCs

**FIGURE 3:**
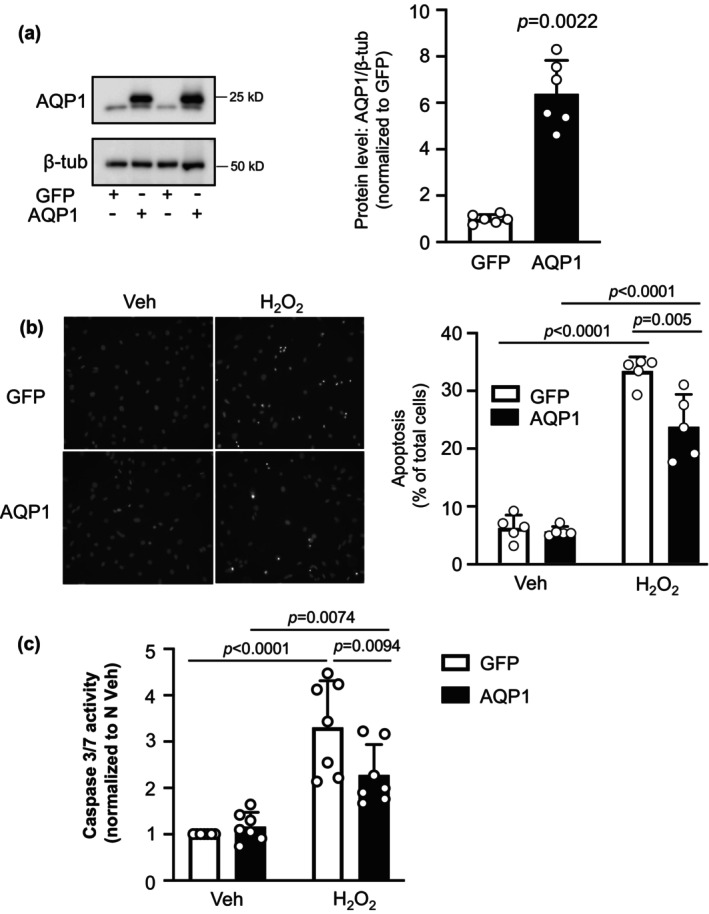
Effect of increasing AQP1 on apoptosis in pulmonary arterial smooth muscle cells (PASMCs) from control rats. (a) Representative blots show AQP1 protein levels in PASMCs infected with adenovirus containing wild‐type AQP1 (AdAQP1) or green fluorescence protein (AdGFP). Bar and scatter plot graphs represent mean ± SD values and individual values (*n* = 6 rats per group) for AQP1 protein levels. Significance determined by *t* test. (b, c) Bar and scatter graphs represent mean ± SD values and individual values for apoptosis measured via (b) Hoescht staining (*n* = 5 rats per group) and (c) caspase 3/7 activity assay (*n* = 7 rats per group) in PASMCs from control rats infected with AdGFP or AdAQP1 and challenged with vehicle (Veh; PBS) or H_2_O_2_ (500 μM; 24 h). Values for apoptosis are presented as percent of total cells while values for caspase 3/7 activity are normalized to AdGFP Veh cells. Significance assessed by two‐way anova with Holm‐Sidak post hoc test. For (b), interaction *p* = 0.0072. For (c), interaction *p* = 0.0173.

Since reducing AQP1 levels in SuHx restored susceptibility to an apoptotic stimulus, we next tested whether increasing AQP1 levels in control cells could confer resistance to apoptosis. Using an adenovirus encoding wild‐type AQP1, we were able to increase AQP1 protein levels in control PASMCs (Figure 3a). In these experiments, a lower band was visible in all lanes, corresponding to native AQP1. In samples infected with AdAQP1, a second band of slightly higher weight was clearly visible, corresponding to the expressed HA‐tagged AQP1. Increasing AQP1 protein levels in control cells had no significant effect on percent of cells undergoing apoptosis (Figure 3b) or caspase 3/7 activity (Figure 3c) at baseline. Stimulation with H_2_O_2_ increased both apoptosis (Figure 3b) and caspase 3/7 activity (Figure 3c) in cells infected with AdGFP, as expected, whereas increasing AQP1 levels with AdAQP1 suppressed but did not completely prevent H_2_O_2_ from inducing apoptosis or increasing caspase 3/7 activity.

### 3.4 Effects of H_2_O_2_ on BAX/Bcl‐2 expression in normoxic and SuHx PASMCs

**FIGURE 4:**
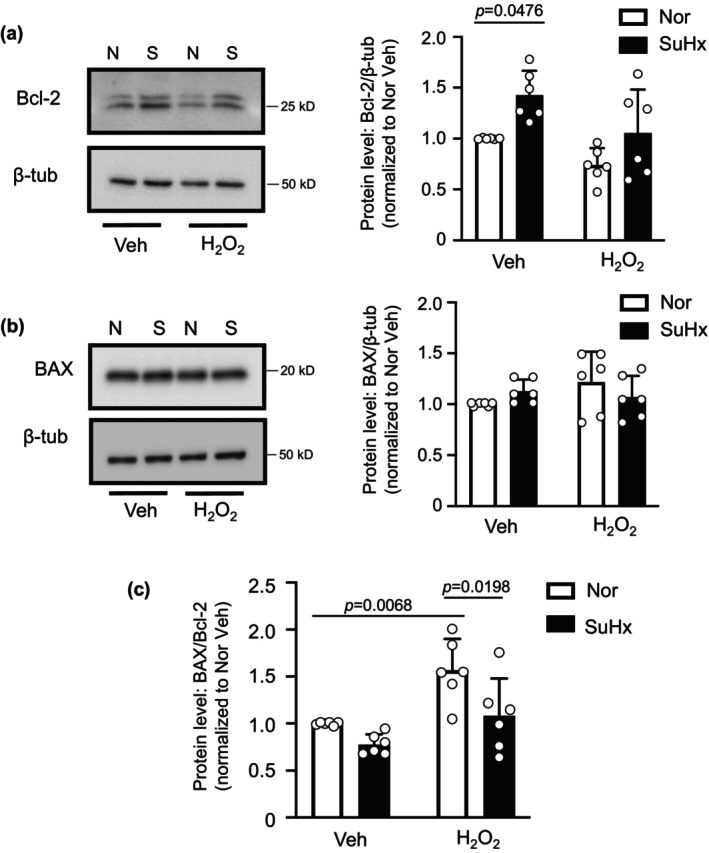
BAX and Bcl‐2 levels in pulmonary arterial smooth muscle cells (PASMCs) from normoxic (N) and Su5416+hypoxia (S) rats. (a) Representative blots show Bcl‐2 protein levels in PASMCs from N and S rats treated with vehicle (Veh; PBS) or H_2_O_2_ (500 μM; 24 h). Bar and scatter plot graphs represent mean ± SD values and individual values for Bcl‐2 protein levels. Significance assessed by two‐way anova with Holm‐Sidak post hoc test. Interaction *p* = 0.0178. (b) Representative blots show BAX protein levels in PASMCs from N and SuHx rats treated with vehicle (Veh; PBS) or H_2_O_2_ (500 μM; 24 h). Bar and scatter plot graphs represent mean ± SD values and individual values for BAX protein levels. Significance assessed by two‐way anova; all values ns. (c) Bar and scatter plot graphs represent mean ± SD values and individual values for the ratio of BAX/Bcl‐2 protein levels. For all experiments, *n* = 6 rats per group. Significance assessed by two‐way anova with Holm‐Sidak post hoc test. Interaction *p* = 0.2427.

To begin to assess the mechanism by which AQP1 protein expression might control caspase 3/7 activity and apoptosis, we measured the levels of Bcl‐2 family proteins: Bcl‐2 and BAX. During apoptosis, levels of the anti‐apoptotic protein, Bcl‐2, typically decrease, allowing accumulation and/or translocation of pro‐apoptotic BAX to mitochondria, where it forms pores through which cytochrome C fragments are released into the cytosol and activate caspase 3. In vehicle‐treated cells, Bcl‐2 protein levels were significantly higher in PASMCs from SuHx rats compared to controls (Figure 4a). Challenge with H_2_O_2_ reduced Bcl‐2 protein levels in cells from normoxic animals, although the difference did not reach statistical significance (*p* = 0.1813). Bcl‐2 levels in SuHx cells challenged with H_2_O_2_ were lower but not statistically significantly different from the values measured in SuHx cells challenged with vehicle. Total BAX protein levels were similar in normoxic and SuHx cells at baseline and did not change with exposure to H_2_O_2_ in either cell type. The ratio of BAX/Bcl‐2 expression is a surrogate for apoptotic susceptibility. At baseline (vehicle treatment), the BAX/Bcl‐2 ratio was not different in normoxic and SuHx cells. However, because of the trends in Bcl‐2 and BAX protein levels, H_2_O_2_ significantly increased the ratio of BAX/Bcl‐2 expression in cells from normoxic rats, whereas the ratio was significantly lower in H_2_O_2_‐challenged cells from SuHx rats (Figure 4c).

### 3.5 Effect of AQP1 depletion on Bcl‐2 and BAX protein levels in SuHx PASMCs

**FIGURE 5:**
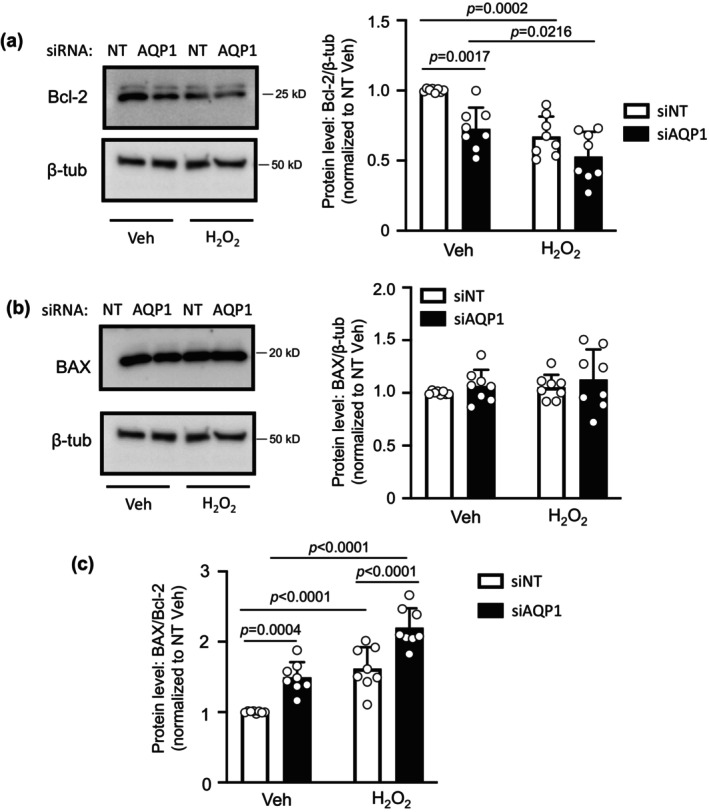
Effect of depleting AQP1 protein on Bcl‐2 and BAX protein levels in pulmonary arterial smooth muscle cells (PASMCs) from Su5416+hypoxia (SuHx) rats. (a) Representative blots show Bcl‐2 protein levels in PASMCs from SuHx rats transfected with siRNA targeted to AQP1 (siAQP1) or a nontargeting siRNA (siNT) and treated with vehicle (Veh; PBS) or H_2_O_2_ (500 μM; 24 h). Bar and scatter plot graphs represent mean ± SD values and individual values for Bcl‐2 protein levels. Significance assessed by two‐way anova with Holm‐Sidak post hoc test. Interaction *p* = 0.1861. (b) Representative blots show BAX protein levels in PASMCs from SuHx rats treated with Veh or H_2_O_2_. Bar and scatter plot graphs represent mean ± SD values and individual values for BAX protein levels. Significance assessed by two‐way anova with Holm‐Sidak post hoc test. All values ns. (c) Bar and scatter plot graphs represent mean ± SD values and individual values for the ratio of BAX/Bcl‐2 protein levels. For all experiments, *n* = 8 rats per group. Significance assessed by two‐way anova with Holm‐Sidak post hoc test. Interaction *p* = 0.5906.

Given our finding that the BAX/Bcl‐2 ratio following challenge with H_2_O_2_ was significantly reduced in SuHx PASMCs, we next explored whether reducing AQP1 levels in PASMCs from SuHx could restore the effect of H_2_O_2_ on the BAX/Bcl‐2 ratio. Depletion of AQP1 in PASMCs from SuHx rats significantly reduced basal Bcl‐2 protein levels (Figure 5a). Application of H_2_O_2_ reduced Bcl‐2 protein levels in SuHx infected with siNT, an effect that was significantly amplified when AQP1 was depleted. Total BAX protein levels remained fairly constant across groups (siNT and siAQP1, with and without H_2_O_2_) (Figure 5b). Because of the reductions in Bcl‐2, depletion of AQP1 significantly increased the ratio of BAX/Bcl‐2 in vehicle‐treated SuHx PASMCs at baseline, and significantly enhanced the ability of H_2_O_2_ to increase the ratio of BAX/Bcl‐2 protein (Figure 5c).

### 3.6 Effect of increasing AQP1 protein levels on Bcl‐2 and BAX in control PASMCs

**FIGURE 6:**
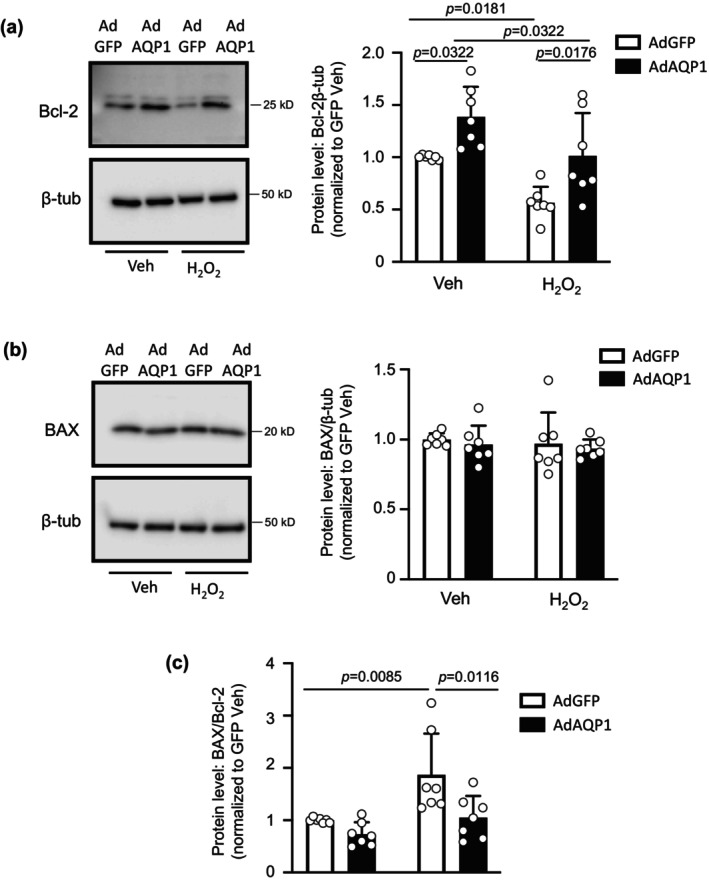
Effect of increasing AQP1 protein levels on Bcl‐2 and BAX expression in control pulmonary arterial smooth muscle cells (PASMCs). (a) Representative blots show Bcl‐2 protein levels in PASMCs from control rats infected with adenovirus containing wild‐type AQP1 (AdAQP1) or green fluorescent protein (AdGFP) and treated with vehicle (Veh; PBS) or H_2_O_2_ (500 μM; 24 h). Bar and scatter plot graphs represent mean ± SD values and individual values for Bcl‐2 protein levels. Significance assessed by two‐way anova with Holm‐Sidak post hoc test. Interaction *p* = 0.7422. (b) Representative blots show BAX protein levels in PASMCs from control rats with forced expression of AQP1 or GFP and treated with Veh or H_2_O_2_. Bar and scatter plot graphs represent mean ± SD values and individual values for BAX protein levels. Significance assessed by two‐way anova with Holm‐Sidak post hoc test. All values ns. (c) Bar and scatter plot graphs represent mean ± SD values and individual values for the ratio of BAX/Bcl‐2 protein levels. Significance assessed by two‐way anova with Holm‐Sidak post hoc test. Interaction *p* = 0.1291. For all experiments, *n* = 7 rats per group.

In a final set of experiments, we tested whether forced expression of AQP1 was sufficient to alter Bcl‐2 and BAX protein expression. In PASMCs from control rats where we enhanced expression of AQP1, baseline Bcl‐2 protein levels were higher than in cells expressing AdGFP, and the H_2_O_2_‐induced decrease was attenuated (Figure 6a). Increasing AQP1 levels, in the absence or presence of H_2_O_2_, had no effect on total BAX protein levels (Figure 6b). The increase in Bcl‐2 levels achieved with AdAQP1 was not sufficient to significantly reduce the BAX/Bcl‐2 ratio at baseline; however, increasing AQP1 resulted in a significant reduction in the BAX/Bcl‐2 ratio in response to H_2_O_2_ compared to H_2_O_2_‐challenged cells infected with AdGFP (Figure 6c).

## 4 DISCUSSION

In the current study, we tested the role of AQP1 in modulating apoptosis in PASMCs from control cells and in cells from a robust model of PH. We found that elevated levels of AQP1 in PASMCs from pulmonary hypertensive rats were necessary for resistance to apoptosis and that some level of apoptosis resistance could be conferred simply by increasing expression of AQP1 in PASMCs from control rats. Moreover, in exploring the downstream pathways involved, we found AQP1 levels influence the expression of Bcl‐2, with enhanced AQP1 levels corresponding to increased Bcl‐2 expression, leading to reductions in the ratio of BAX to Bcl‐2 that are typically associated with apoptosis resistance.

It has been widely reported that pulmonary vascular cells from patients with PAH (Dromparis et al., 2010; Lampron et al., 2020; Meloche et al., 2015) and from pre‐clinical models of PH (McMurtry et al., 2005; Meloche et al., 2015) are resistant to apoptosis. We confirmed these findings in PASMCs from the SuHx rat model of PH, which recapitulates several of the features of human PAH. Using two complementary methods to assess apoptosis, morphology via Hoechst staining and caspase 3/7 activity, we showed cells from SuHx were resistant to apoptosis induced by H_2_O_2_. Preliminary experiments demonstrated that suppression of apoptosis in these cells was not restricted to a single apoptotic stimulus, as PASMCs from SuHx rats also exhibited reduced apoptosis in response to staurosporine (Figure S1).

An interesting finding from our study was that there was no difference in apoptosis or caspase 3/7 activity at baseline between cells from normoxic and SuHx rats, despite conditions that should produce endogenous pro‐apoptotic stimuli, including mitochondrial dysfunction, ER stress, and hyperproliferation (Chen et al., 2018; Huetsch et al., 2019). The lack of increase in baseline apoptosis under these conditions suggests that during PH, PASMCs develop mechanisms to evade activation of executioner caspases. In many types of cancer cells, AQP1 protein levels are elevated and inversely correlated with susceptibility to apoptosis (Lehnerdt et al., 2015; Moosavi & Elham, 2020; Simone et al., 2018; Tomita et al., 2017), although in other tumor cells (i.e., mesothelioma) AQP1 and apoptosis were not associated (Klebe et al., 2015). AQP1 expression was also inversely correlated with apoptosis in aortic endothelial (Sada et al., 2016) and lens epithelial (Zheng et al., 2016) cells. Since SuHx cells also exhibit increased AQP1 expression (Yun et al., 2021) (Figure 1) we next tested whether the increased AQP1 levels in SuHx PASMCs were essential for apoptosis resistance. Using siRNA approaches, we showed that reducing the amount of AQP1 in SuHx PASMCs increased apoptosis at baseline and restored the response to H_2_O_2_. These results are consistent with effects reported for hypoxia‐induced apoptosis in PASMCs (Liu et al., 2019). Although enhanced levels of AQP1 appear to be required to maintain apoptosis resistance in SuHx PASMCs, our results indicate that simply increasing AQP1 levels was not sufficient to fully promote apoptosis resistance. While a reduction in H_2_O_2_‐induced apoptosis and caspase 3/7 activity was noted when AQP1 levels were elevated in control PASMCs, the effect was modest, indicating that other factors are also participating in the development of apoptosis resistance during PH. These factors could include increased levels of survivin (McMurtry et al., 2005) or FOXM1 (Bourgeois et al., 2018), both of which have been associated with apoptosis resistance in PAH. Whether these pathways interact with AQP1 is currently unknown.

Apoptosis can be triggered via intrinsic or extrinsic pathways. In either case, a common component of the pathways is the release of fragmented cytochrome C from pores in the mitochondrial membrane formed by the pro‐apoptotic Bcl‐2 family member, BAX (Antonsson et al., 1997; Lalier et al., 2007). Cytochrome C fragments released into the cytosol activate caspase 3, the executioner caspase, to induce apoptosis. Normally found in the cytosol, BAX activation and translocation to the mitochondria is antagonized by interaction with the pro‐apoptotic protein, Bcl‐2 (Antonsson et al., 1997). Thus, the relative ratio of BAX to Bcl‐2 protein can dictate cellular susceptibility to apoptosis, with lower BAX/Bcl‐2 ratios associated with reduced apoptosis. In normoxic PASMCs, in response to H_2_O_2_, the increase in the ratio of BAX/Bcl‐2 is primarily driven by lower Bcl‐2 expression. In PASMCs from SuHx rats, however, there is a trend toward lower BAX/Bcl‐2 at baseline and a reduced increase in the ratio in response to H_2_O_2_. The reduced BAX/Bcl‐2 ratio in SuHx cells also appears to be driven primarily by increased Bcl‐2 expression, a finding consistent with various tumor cells, where Bcl‐2 expression is enhanced, leading to apoptosis resistance (Kaloni et al., 2023). Thus, AQP1‐mediated increase in Bcl‐2 expression in SuHx cells provides a mechanism for resisting apoptosis. These results are consistent with findings reported in squamous cell carcinoma, where AQP1 protein expression was observed in cells with high Bcl‐2 expression (Lehnerdt et al., 2015).

In contrast to what has often been reported for other cell types (Han et al., 1996; Lalier et al., 2007), we did not observe a change in BAX levels with apoptotic stimulus. While this result was somewhat surprising, some reports have noted that apoptosis is not always accompanied by an increase in BAX (Benito et al., 1996). Since we measured total BAX levels, it is possible that observed changes in apoptosis were driven primarily by increased or decreased interaction with Bcl‐2, preventing or enhancing, respectively, translocation and formation of mitochondrial pores. Another possibility is that changes in BAX expression occur at an earlier time point than that at which we collected proteins for analysis. Assessing these possibilities will require further experimentation.

While our results demonstrate that AQP1 levels can modulate Bcl‐2 expression, the exact mechanism remains unclear. Several reports suggest that β‐catenin may play a role. For example, in endothelial cells β‐catenin promoted cell survival by upregulating Bcl‐2, thus inhibiting BAX activation (Tajadura et al., 2020). Similarly, in myocardial and intestinal epithelial cells, enhancing β‐catenin increased Bcl‐2 expression (Kaga et al., 2006; Mezhybovska et al., 2006). We previously reported that AQP1 regulates β‐catenin protein levels (Yun et al., 2017), through a mechanism involving the C‐terminal tail region. Given the plethora of data showing β‐catenin regulates Bcl‐2 expression, it seems plausible that β‐catenin may also be the link between AQP1 and Bcl‐2 in PASMCs, although additional experiments will be needed to test this possibility.

Another potential mechanism by which AQP1 could modulate apoptosis is via water transport. In the earliest stages of apoptosis, one of the most highly conserved events is cell shrinkage due to water loss or apoptotic volume loss. Consistent with this hypothesis, in granulosa cells, blockade of AQP1 water permeability with mercury prevents cell shrinkage and caspase activation (Jablonski et al., 2004). However, in PASMCs, we observed the opposite effect, with loss of AQP1 promoting apoptosis and activation of caspase 3/7. Whether this reflects the fact that AQP1 levels were reduced, but not completely eliminated, allowing for sufficient water transport to permit cell shrinkage, is unknown. Importantly, AQP1 was the only aquaporin identified in granulosa cells, whereas PASMCs also express aquaporins 4 and 7 (Leggett et al., 2012), which may also contribute to water permeability and facilitate apoptotic volume loss to allow apoptosis to proceed when AQP1 abundance is reduced.

AQP1 has also been suggested to facilitate entry of H_2_O_2_ into vascular cells (Al Ghouleh et al., 2013). In this case, depletion of AQP1 could be speculated to prevent apoptosis induced by the application of exogenous H_2_O_2_ by reducing H_2_O_2_ levels within the cell. While our experiments cannot entirely rule out this possibility, initial experiments revealed that SuHx cells were also resistant to apoptosis in response to staurosporine, a broad kinase inhibitor (Figure S1), and preliminary studies showed depletion of AQP1 in SuHx PASMCs significantly increased both basal and staurosporine‐induced apoptosis (Figure S2). These results would suggest that reducing the speed at which H_2_O_2_ enters the cell is not the main mechanism by which AQP1 confers resistance to apoptosis.

In summary, we found that similar to vascular cells from patients with PAH (Dromparis et al., 2010; Lampron et al., 2020; Meloche et al., 2015), PASMCs from the SuHx rat model of PH are resistant to apoptosis via a mechanism involving AQP1‐mediated increases in Bcl‐2 expression. While necessary for apoptosis resistance observed in these cells, increased AQP1 levels in and of themselves are not sufficient to fully drive apoptosis resistance, suggesting other factors also contribute. These early results provide a mechanism by which AQP1 can regulate PASMC fate and suggest that further investigation could provide additional clues as to whether AQP1‐mediated apoptosis resistance contributes to PAH development or progression and whether AQP1 might be a suitable target for therapy.

## CONFLICT OF INTEREST STATEMENT

None.

## ETHICS STATEMENT

The experimental protocols used in this study conform to ARRIVE guidelines and the Guide for the Care and Use of Laboratory Animals (National Institutes of Health) and were approved by the Johns Hopkins Institutional Animal Care and Use Committee (protocol RA20M171). Johns Hopkins University is accredited by the Association for Assessment and Accreditation of Laboratory Animal Care International.

## Supporting information


Figures S1‐S2.

## Data Availability

The data that support the findings of this study are available from the corresponding author upon reasonable request.

## References

[phy216156-bib-0001] Agre, P. , King, L. S. , Yasui, M. , Guggino, W. B. , Ottersen, O. P. , Fujiyoshi, Y. , Engel, A. , & Nielsen, S. (2002). Aquaporin water channels – From atomic structure to clinical medicine. The Journal of Physiology, 542, 3–16. 12096044 10.1113/jphysiol.2002.020818 PMC2290382

[phy216156-bib-0002] Al Ghouleh, I. , Frazziano, G. , Rodriguez, A. I. , Csanyi, G. , Maniar, S. , St Croix, C. M. , Kelley, E. E. , Egana, L. A. , Song, G. J. , Bisello, A. , Lee, Y. J. , & Pagano, P. J. (2013). Aquaporin 1, Nox1, and Ask1 mediate oxidant‐induced smooth muscle cell hypertrophy. Cardiovascular Research, 97, 134–142. 22997161 10.1093/cvr/cvs295 PMC3527765

[phy216156-bib-0003] Antonsson, B. , Conti, F. , Ciavatta, A. , Montessuit, S. , Lewis, S. , Martinou, I. , Bernasconi, L. , Bernard, A. , Mermod, J. J. , Mazzei, G. , Maundrell, K. , Gambale, F. , Sadoul, R. , & Martinou, J. C. (1997). Inhibition of BAX channel‐forming activity by Bcl‐2. Science, 277, 370–372. 9219694 10.1126/science.277.5324.370

[phy216156-bib-0004] Benito, A. , Silva, M. , Grillot, D. , Nunez, G. , & Fernandez‐Luna, J. L. (1996). Apoptosis induced by erythroid differentiation of human leukemia cell lines is inhibited by Bcl‐XL. Blood, 87, 3837–3843. 8611710

[phy216156-bib-0005] Bourgeois, A. , Lambert, C. , Habbout, K. , Ranchoux, B. , Paquet‐Marceau, S. , Trinh, I. , Breuils‐Bonnet, S. , Paradis, R. , Nadeau, V. , Paulin, R. , Provencher, S. , Bonnet, S. , & Boucherat, O. (2018). FOXM1 promotes pulmonary artery smooth muscle cell expansion in pulmonary arterial hypertension. Journal of Molecular Medicine (Berlin, Germany), 96, 223–235. 29290032 10.1007/s00109-017-1619-0

[phy216156-bib-0006] Chen, K. H. , Dasgupta, A. , Lin, J. , Potus, F. , Bonnet, S. , Iremonger, J. , Fu, J. , Mewburn, J. , Wu, D. , Dunham‐Snary, K. , Theilmann, A. L. , Jing, Z. C. , Hindmarch, C. , Ormiston, M. L. , Lawrie, A. , & Archer, S. L. (2018). Epigenetic dysregulation of the dynamin‐related protein 1 binding partners MiD49 and MiD51 increases mitotic mitochondrial fission and promotes pulmonary arterial hypertension: Mechanistic and therapeutic implications. Circulation, 138, 287–304. 29431643 10.1161/CIRCULATIONAHA.117.031258 PMC6050130

[phy216156-bib-0007] Ding, T. , Zhou, Y. , Sun, K. , Jiang, W. , Li, W. , Liu, X. , Tian, C. , Li, Z. , Ying, G. , Fu, L. , Gu, F. , Li, W. , & Ma, Y. (2013). Knockdown a water channel protein, aquaporin‐4, induced glioblastoma cell apoptosis. PLoS One, 8, e66751. 23950863 10.1371/journal.pone.0066751 PMC3741385

[phy216156-bib-0008] Dromparis, P. , Sutendra, G. , & Michelakis, E. D. (2010). The role of mitochondria in pulmonary vascular remodeling. Journal of Molecular Medicine (Berlin, Germany), 88, 1003–1010. 20734021 10.1007/s00109-010-0670-x

[phy216156-bib-0009] Edlich, F. (2018). BCL‐2 proteins and apoptosis: Recent insights and unknowns. Biochemical and Biophysical Research Communications, 500, 26–34. 28676391 10.1016/j.bbrc.2017.06.190

[phy216156-bib-0010] Han, J. , Sabbatini, P. , Perez, D. , Rao, L. , Modha, D. , & White, E. (1996). The E1B 19K protein blocks apoptosis by interacting with and inhibiting the p53‐inducible and death‐promoting BAX protein. Genes & Development, 10, 461–477. 8600029 10.1101/gad.10.4.461

[phy216156-bib-0011] Hoque, M. O. , Soria, J. C. , Woo, J. , Lee, T. , Lee, J. , Jang, S. J. , Upadhyay, S. , Trink, B. , Monitto, C. , Desmaze, C. , Mao, L. , Sidransky, D. , & Moon, C. (2006). Aquaporin 1 is overexpressed in lung cancer and stimulates NIH‐3T3 cell proliferation and anchorage‐independent growth. The American Journal of Pathology, 168, 1345–1353. 16565507 10.2353/ajpath.2006.050596 PMC1606549

[phy216156-bib-0012] Hua, T. , Robitaille, M. , Roberts‐Thomson, S. J. , & Monteith, G. R. (2023). The intersection between cysteine proteases, Ca(2+) signalling and cancer cell apoptosis. Biochimica et Biophysica Acta (BBA)‐Molecular Cell Research, 1870, 119532. 37393017 10.1016/j.bbamcr.2023.119532

[phy216156-bib-0013] Huetsch, J. , Yun, X. , Jiang, H. , & Shimoda, L. (2019). ER stress induced apoptosis of smooth muscle in pulmonary hypertension is regulated by the sodium‐hydrogen exchanger. The FASEB Journal, 33, 550.1.

[phy216156-bib-0014] Huetsch, J. C. , Jiang, H. , Larrain, C. , & Shimoda, L. A. (2016). The Na+/H+ exchanger contributes to increased smooth muscle proliferation and migration in a rat model of pulmonary arterial hypertension. Physiological Reports, 4, e12729. 26997630 10.14814/phy2.12729 PMC4823603

[phy216156-bib-0015] Jablonski, E. M. , Webb, A. N. , McConnell, N. A. , Riley, M. C. , & Hughes, F. M., Jr. (2004). Plasma membrane aquaporin activity can affect the rate of apoptosis but is inhibited after apoptotic volume decrease. American Journal of Physiology. Cell Physiology, 286, C975–C985. 14644770 10.1152/ajpcell.00180.2003

[phy216156-bib-0016] Jurasz, P. , Courtman, D. , Babaie, S. , & Stewart, D. J. (2010). Role of apoptosis in pulmonary hypertension: From experimental models to clinical trials. Pharmacology & Therapeutics, 126, 1–8. 20117135 10.1016/j.pharmthera.2009.12.006

[phy216156-bib-0017] Kaga, S. , Zhan, L. , Altaf, E. , & Maulik, N. (2006). Glycogen synthase kinase‐3beta/beta‐catenin promotes angiogenic and anti‐apoptotic signaling through the induction of VEGF, Bcl‐2 and survivin expression in rat ischemic preconditioned myocardium. Journal of Molecular and Cellular Cardiology, 40, 138–147. 16288908 10.1016/j.yjmcc.2005.09.009

[phy216156-bib-0018] Kaloni, D. , Diepstraten, S. T. , Strasser, A. , & Kelly, G. L. (2023). BCL‐2 protein family: Attractive targets for cancer therapy. Apoptosis, 28, 20–38. 36342579 10.1007/s10495-022-01780-7 PMC9950219

[phy216156-bib-0019] Klebe, S. , Griggs, K. , Cheng, Y. , Driml, J. , Henderson, D. W. , & Reid, G. (2015). Blockade of aquaporin 1 inhibits proliferation, motility, and metastatic potential of mesothelioma in vitro but not in an in vivo model. Disease Markers, 2015, 286719. 25821338 10.1155/2015/286719 PMC4364038

[phy216156-bib-0020] Lai, N. , Lade, J. , Leggett, K. , Yun, X. , Baksh, S. , Chau, E. , Crow, M. T. , Sidhaye, V. , Wang, J. , & Shimoda, L. A. (2014). The aquaporin 1 C‐terminal tail is required for migration and growth of pulmonary arterial myocytes. American Journal of Respiratory Cell and Molecular Biology, 50, 1010–1020. 24328827 10.1165/rcmb.2013-0374OC PMC5946682

[phy216156-bib-0021] Lalier, L. , Cartron, P. F. , Juin, P. , Nedelkina, S. , Manon, S. , Bechinger, B. , & Vallette, F. M. (2007). BAX activation and mitochondrial insertion during apoptosis. Apoptosis, 12, 887–896. 17453158 10.1007/s10495-007-0749-1

[phy216156-bib-0022] Lampron, M. C. , Vitry, G. , Nadeau, V. , Grobs, Y. , Paradis, R. , Samson, N. , Tremblay, E. , Boucherat, O. , Meloche, J. , Bonnet, S. , Provencher, S. , Potus, F. , & Paulin, R. (2020). PIM1 (moloney murine leukemia provirus integration site) inhibition decreases the nonhomologous end‐joining DNA damage repair signaling pathway in pulmonary hypertension. Arteriosclerosis, Thrombosis, and Vascular Biology, 40, 783–801. 31969012 10.1161/ATVBAHA.119.313763

[phy216156-bib-0023] Lee, Y. G. , Yang, N. , Chun, I. , Porazzi, P. , Carturan, A. , Paruzzo, L. , Sauter, C. T. , Guruprasad, P. , Pajarillo, R. , & Ruella, M. (2023). Apoptosis: A Janus bifrons in T‐cell immunotherapy. Journal for Immunotherapy of Cancer, 11, e005967. 37055217 10.1136/jitc-2022-005967 PMC10106075

[phy216156-bib-0024] Leggett, K. , Maylor, J. , Undem, C. , Lai, N. , Lu, W. , Schweitzer, K. S. , King, L. S. , Myers, A. C. , Sylvester, J. T. , Sidhaye, V. K. , & Shimoda, L. A. (2012). Hypoxia‐induced migration in pulmonary arterial smooth muscle cells requires calcium‐dependent upregulation of aquaporin 1. American Journal of Physiology. Lung Cellular and Molecular Physiology, 303(4), L343–L353. 22683574 10.1152/ajplung.00130.2012 PMC3423828

[phy216156-bib-0025] Lehnerdt, G. F. , Bachmann, H. S. , Adamzik, M. , Panic, A. , Koksal, E. , Weller, P. , Lang, S. , Schmid, K. W. , Siffert, W. , & Bankfalvi, A. (2015). AQP1, AQP5, Bcl‐2 and p16 in pharyngeal squamous cell carcinoma. The Journal of Laryngology and Otology, 129, 580–586. 26074259 10.1017/S002221511500119X

[phy216156-bib-0026] Liu, M. , Liu, Q. , Pei, Y. , Gong, M. , Cui, X. , Pan, J. , Zhang, Y. , Liu, Y. , Liu, Y. , Yuan, X. , Zhou, H. , Chen, Y. , Sun, J. , Wang, L. , Zhang, X. , Wang, R. , Li, S. , Cheng, J. , Ding, Y. , … Yuan, Y. (2019). Aqp‐1 gene knockout attenuates hypoxic pulmonary hypertension of mice. Arteriosclerosis, Thrombosis, and Vascular Biology, 39, 48–62. 30580569 10.1161/ATVBAHA.118.311714

[phy216156-bib-0027] Masri, F. A. , Xu, W. , Comhair, S. A. , Asosingh, K. , Koo, M. , Vasanji, A. , Drazba, J. , Anand‐Apte, B. , & Erzurum, S. C. (2007). Hyperproliferative apoptosis‐resistant endothelial cells in idiopathic pulmonary arterial hypertension. American Journal of Physiology. Lung Cellular and Molecular Physiology, 293, L548–L554. 17526595 10.1152/ajplung.00428.2006

[phy216156-bib-0028] McMurtry, M. S. , Archer, S. L. , Altieri, D. C. , Bonnet, S. , Haromy, A. , Harry, G. , Puttagunta, L. , & Michelakis, E. D. (2005). Gene therapy targeting survivin selectively induces pulmonary vascular apoptosis and reverses pulmonary arterial hypertension. The Journal of Clinical Investigation, 115, 1479–1491. 15931388 10.1172/JCI23203 PMC1136986

[phy216156-bib-0029] Meloche, J. , Le Guen, M. , Potus, F. , Vinck, J. , Ranchoux, B. , Johnson, I. , Antigny, F. , Tremblay, E. , Breuils‐Bonnet, S. , Perros, F. , Provencher, S. , & Bonnet, S. (2015). miR‐223 reverses experimental pulmonary arterial hypertension. American Journal of Physiology. Cell Physiology, 309, C363–C372. 26084306 10.1152/ajpcell.00149.2015

[phy216156-bib-0030] Mezhybovska, M. , Wikstrom, K. , Ohd, J. F. , & Sjolander, A. (2006). The inflammatory mediator leukotriene D4 induces beta‐catenin signaling and its association with antiapoptotic Bcl‐2 in intestinal epithelial cells. The Journal of Biological Chemistry, 281, 6776–6784. 16407243 10.1074/jbc.M509999200

[phy216156-bib-0031] Moldoveanu, T. , & Czabotar, P. E. (2020). BAX, BAK, and BOK: A coming of age for the BCL‐2 family effector proteins. Cold Spring Harbor Perspectives in Biology, 12, a036319. 31570337 10.1101/cshperspect.a036319 PMC7111251

[phy216156-bib-0032] Moosavi, M. S. , & Elham, Y. (2020). Aquaporins 1, 3 and 5 in different tumors, their expression, prognosis value and role as new therapeutic targets. Pathology Oncology Research, 26, 615–625. 30927206 10.1007/s12253-019-00646-9

[phy216156-bib-0033] Morrell, N. W. , Yang, X. , Upton, P. D. , Jourdan, K. B. , Morgan, N. , Sheares, K. K. , & Trembath, R. C. (2001). Altered growth responses of pulmonary artery smooth muscle cells from patients with primary pulmonary hypertension to transforming growth factor‐β1 and bone morphogenetic proteins. Circulation, 104, 790–795. 11502704 10.1161/hc3201.094152

[phy216156-bib-0034] Sada, K. , Nishikawa, T. , Kukidome, D. , Yoshinaga, T. , Kajihara, N. , Sonoda, K. , Senokuchi, T. , Motoshima, H. , Matsumura, T. , & Araki, E. (2016). Hyperglycemia induces cellular hypoxia through production of mitochondrial ROS followed by suppression of aquaporin‐1. PLoS One, 11, e0158619. 27383386 10.1371/journal.pone.0158619 PMC4934928

[phy216156-bib-0035] Schuoler, C. , Haider, T. J. , Leuenberger, C. , Vogel, J. , Ostergaard, L. , Kwapiszewska, G. , Kohler, M. , Gassmann, M. , Huber, L. C. , & Brock, M. (2017). Aquaporin 1 controls the functional phenotype of pulmonary smooth muscle cells in hypoxia‐induced pulmonary hypertension. Basic Research in Cardiology, 112, 30. 28409279 10.1007/s00395-017-0620-7

[phy216156-bib-0036] Shu, C. , Shu, Y. , Gao, Y. , Chi, H. , & Han, J. (2019). Inhibitory effect of AQP1 silencing on adhesion and angiogenesis in ectopic endometrial cells of mice with endometriosis through activating the Wnt signaling pathway. Cell Cycle, 18, 2026–2039. 31251110 10.1080/15384101.2019.1637202 PMC6681775

[phy216156-bib-0037] Simone, L. , Gargano, C. D. , Pisani, F. , Cibelli, A. , Mola, M. G. , Frigeri, A. , Svelto, M. , & Nicchia, G. P. (2018). Aquaporin‐1 inhibition reduces metastatic formation in a mouse model of melanoma. Journal of Cellular and Molecular Medicine, 22, 904–912. 29044946 10.1111/jcmm.13378 PMC5783831

[phy216156-bib-0038] Stenmark, K. R. , Frid, M. G. , Graham, B. B. , & Tuder, R. M. (2018). Dynamic and diverse changes in the functional properties of vascular smooth muscle cells in pulmonary hypertension. Cardiovascular Research, 114, 551–564. 29385432 10.1093/cvr/cvy004 PMC5852549

[phy216156-bib-0039] Tajadura, V. , Hansen, M. H. , Smith, J. , Charles, H. , Rickman, M. , Farrell‐Dillon, K. , Claro, V. , Warboys, C. , & Ferro, A. (2020). β‐catenin promotes endothelial survival by regulating eNOS activity and flow‐dependent anti‐apoptotic gene expression. Cell Death & Disease, 11, 493. 32606304 10.1038/s41419-020-2687-6 PMC7326989

[phy216156-bib-0040] Tomita, Y. , Dorward, H. , Yool, A. J. , Smith, E. , Townsend, A. R. , Price, T. J. , & Hardingham, J. E. (2017). Role of aquaporin 1 signalling in cancer development and progression. International Journal of Molecular Sciences, 18, 299. 28146084 10.3390/ijms18020299 PMC5343835

[phy216156-bib-0041] Wu, Z. , Li, S. , Liu, J. , Shi, Y. , Wang, J. , Chen, D. , Luo, L. , Qian, Y. , Huang, X. , & Wang, H. (2015). RNAi‐mediated silencing of AQP1 expression inhibited the proliferation, invasion and tumorigenesis of osteosarcoma cells. Cancer Biology & Therapy, 16, 1332–1340. 26176849 10.1080/15384047.2015.1070983 PMC4623513

[phy216156-bib-0042] Yu, Y. , Fantozzi, I. , Remillard, C. V. , Landsberg, J. W. , Kunichika, N. , Platoshyn, O. , Tigno, D. D. , Thistlethwaite, P. A. , Rubin, L. J. , & Yuan, J. X. (2004). Enhanced expression of transient receptor potential channels in idiopathic pulmonary arterial hypertension. Proceedings of the National Academy of Sciences of the United States of America, 101, 13861–13866. 15358862 10.1073/pnas.0405908101 PMC518765

[phy216156-bib-0043] Yun, X. , Jiang, H. , Lai, N. , Wang, J. , & Shimoda, L. A. (2017). Aquaporin 1‐mediated changes in pulmonary arterial smooth muscle cell migration and proliferation involve β‐catenin. American Journal of Physiology. Lung Cellular and Molecular Physiology, 313, L889–L898. 28798257 10.1152/ajplung.00247.2016 PMC5792177

[phy216156-bib-0044] Yun, X. , Philip, N. M. , Jiang, H. , Smith, Z. , Huetsch, J. , Damarla, M. , Suresh, K. , & Shimoda, L. A. (2021). Upregulation of aquaporin 1 mediates increased migration and proliferation in pulmonary vascular cells from the rat SU5416/hypoxia model of pulmonary hypertension. Frontiers in Physiology, 12, 763444. 34975522 10.3389/fphys.2021.763444 PMC8718640

[phy216156-bib-0045] Zhang, Q. , Lin, L. , Li, W. , Lu, G. , & Li, X. (2019). MiR‐223 inhibitor suppresses proliferation and induces apoptosis of thyroid cancer cells by down‐regulating aquaporin‐1. Journal of Receptor and Signal Transduction Research, 39, 146–153. 31311397 10.1080/10799893.2019.1638403

[phy216156-bib-0046] Zhang, X. , Chen, Y. , Dong, L. , & Shi, B. (2018). Effect of selective inhibition of aquaporin 1 on chemotherapy sensitivity of J82 human bladder cancer cells. Oncology Letters, 15, 3864–3869. 29467903 10.3892/ol.2018.7727 PMC5796269

[phy216156-bib-0047] Zheng, H. H. , Xu, G. X. , Guo, J. , Fu, L. C. , & Yao, Y. (2016). Aquaporin‐1 down regulation associated with inhibiting cell viability and inducing apoptosis of human lens epithelial cells. International Journal of Ophthalmology, 9, 15–20. 26949604 10.18240/ijo.2016.01.03 PMC4768510

