# Supplementary figures and images for: Aquaporin 1 confers apoptosis resistance in pulmonary arterial smooth muscle cells from the SU5416 hypoxia rat model
Source: Physiol Rep. 2024 Aug 22;12(16):e16156. doi: 10.14814/phy2.16156 (PMC11341275; doi:10.14814/phy2.16156)

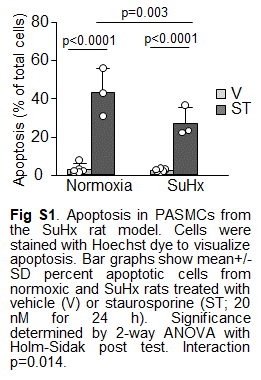


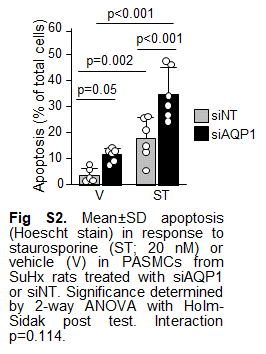

Supplement: Supplementary file 1 — Figures S1‐S2. [file PHY2-12-e16156-s001.docx]
